# Energy Gap-Refractive Index Relations in Perovskites

**DOI:** 10.3390/ma13081917

**Published:** 2020-04-19

**Authors:** Aneer Lamichhane, Nuggehalli M. Ravindra

**Affiliations:** 1 Interdisciplinary Program in Materials Science & Engineering, New Jersey Institute of Technology, Newark, NJ 07102, USA; al593@njit.edu; 2 Department of Physics, New Jersey Institute of Technology, Newark, NJ 07102, USA

**Keywords:** energy gap, refractive index, perovskites

## Abstract

In this study, the energy gap-refractive index relations of perovskites are examined in detail. In general, the properties of perovskites are dependent on the structural reorganization and covalent nature of their octahedral cages. Based on this notion, a simple relation governing the energy gap and the refractive index is proposed for perovskites. The results obtained with this relation are in good accord with the literature values and are consistent with some well-established relations.

## 1. Introduction

Perovskites are materials having the crystal structure of strontium titanate at room temperature ($SrTiO_{3}$) with a general formula for the oxide analogs of $ABX_{3}$, where *A* is a cation, generally a rare-earth- or alkali-type element, *B* is a transition metal cation, and *X* is an oxide or halide anion [1,2,3]. In recent years, there has been a growing interest among material scientists in the study of perovskites [4,5,6]. This is because perovskites exhibit a variety of functions such as piezoelectric, pyroelectric, ferroelectric, photovoltaic cells, LEDs, superconductivity, and topological insulators [7,8,9,10]. Generally, oxide perovskites exhibit good dielectric properties, and halide perovskites show excellent photonic properties [11,12]. Since the discovery of calcium titanium oxide, $CaTiO_{3}$, by Gustav Rose in 1839, the research on perovskites remained dormant until the 21st Century [13,14]. The first paper on lead halide perovskites was published in 1892 [15]. The structure of $CsPbI_{3}$, cesium plumbo iodide, was studied in 1959 [16]. It is only in the last decade that perovskites have gained notoriety as materials for photovoltaic conversion. The paper “Organometal Halide Perovskites as Visible-Light Sensitizers for Photovoltaic Cells” by Kojima and Miyasaka et al. [17] has been the catalyst for the exponential growth of research on perovskite solar cells. Due to their inherent direct energy gap that matches the solar spectrum, halide perovskites continue to perform well as photonic materials [18,19]. Moreover, the crystal structures of perovskites show different polymorphs [20,21,22], which further contribute to significant changes in their dielectric and photonic properties.

It is, therefore, crucial to understand the electronic and optical properties of perovskites to predict the behavior of these functionalities. Such predictions are useful to engineer these materials for various applications. Among several properties, the energy gap and the refractive index are fundamental entities whose correlation is vital for the understanding of the optoelectronic behavior of materials, as well as band-gap engineering [23,24,25,26]. While the threshold wavelength for the absorption of photons in semiconductors is determined by the energy gap, the transparency to incident spectral radiation is quantified by the refractive index. Such a correlation between these two fundamental properties is critical for the determination of the choice of semiconductors for applications in electronics and photonics [27]. Several studies about the relationship between the energy gap and the refractive index have been proposed for semiconductors and examined in the past, yielding various theories in this field [27,28,29]. There has been renewed interest in these studies in recent years [30,31,32,33,34,35,36,37]. While several manuscripts have reported on the studies of the energy gap and refractive index of perovskites [38,39,40,41], Blessing N. Ezealigo et.al. [42] performed a detailed experimental investigation into their research entitled “Method to control the optical properties: Band gap energy of mixed halide Organolead perovskites”, and the results obtained have been interpreted by utilizing the single-oscillator model of Wemple–DiDomenico.

To the best of our knowledge and understanding, a detailed study of the correlations between the refractive index and energy gap for all inorganic perovskites is lacking in the literature. This is the first study of its kind being reported here. A comprehensive study of the fundamental properties such as the energy gap and refractive index is of paramount importance for the study of materials, in particular perovskites, since they are the basis for determining their applications in electronics and photonics. Furthermore, computational frameworks in materials science such as “propnet” [34] require pre-knowledge of the database of these material properties. As materials informatics begins to grow, investigations such as these that relate two fundamental macroscopic properties will pave the way for new applications of perovskites.

## 2. Background

From a fundamental point of view, the refractive index of a material is simply defined as the ratio of the speed of light in a vacuum to that in the material. In general, the refractive index of a material is a function of (a) frequency and (b) doping, although studies in the literature report on the dependence of the refractive index on thickness [43], voids [44], grain boundaries [45], etc. In order to minimize such variation, it is good practice to consider a static refractive index, which is obtained from the time-independent electric field or the field at a zero wave vector. In 1950, Moss [46,47] proposed the general relationship between the energy gap ($E_{g}$) and the refractive index (*n*) as,

(1) $$n^{4}E_{g}=95eV$$

This model is based on Bohr’s atomic model of hydrogen. The assumption was that all energy levels in a solid are scaled down by a factor of $\frac{1}{\epsilon_{\infty }^{2}}$, where $\epsilon_{\infty }$ is the optical dielectric constant satisfying the relation [48],

(2) $$\epsilon_{\infty }=n^{2}$$

Since energy levels in a solid are quite complex and involve band structure theory, this gives rise to a structural restriction on Moss’s relation. Based on the band structure, in 1962, Penn [49] proposed a model for an isotropic semiconductor by modifying Callaway’s approximation of the dielectric constant with the inclusion of the Umklapp process and showed the relation,

(3) $$\epsilon_{\infty }=1+{\left(\hbar \omega_{p}/E_{g}\right)}^{2},$$

where $\omega_{p}$ is the plasma frequency [50]. Almost, in all semiconductors, the trajectories of valence and conduction bands are more or less parallel with each other, at least along the symmetry directions. With these considerations, Gupta and Ravindra [51] proposed that the difference between the Penn gap and the energy gap is constant and showed the linear relationships between the energy gap and the refractive index as [52],

(4) $$n=4.084-0.62E_{g}\left(eV\right)$$

However, Equation (4) puts an upper limit on the refractive index. In order to account for both the structural and the refractive index restriction of the Moss relation and the Ravindra relation, respectively, several empirical relations have been presented by various authors [29,53,54]. Based on the oscillatory theory, Herve and Vandamme [55] presented the relation,

(5) $$n=\sqrt{1+{\left(\frac{A}{E_{g}+B}\right)}^{2}}$$

where A=13.6 eV and B=3.4 eV are the constants. Though this relation is quite superior and agrees satisfactorily for most optoelectronic materials, it has shortcomings for materials of the IV-VI group [56].

In light of these drawbacks, this paper examines the correlations between the energy gap and the refractive index in perovskites. Utilizing the Wemple–DiDomenico single electron oscillator approximation [57] and based on the structure of perovskites, a simple relation is proposed for such ternary systems. Later, this model is compared with the Moss (Equation (1)), Ravindra (Equation (4)), and Herve–Vandamme (Equation (5)) models, as well as the reported experimental values of the refractive indices of perovskites in the literature.

## 3. Theory

The dependence of the refractive index (*n*) on the wavelength (λ) or frequency (ν) has been well described by dispersion relations. The first dispersion relation was developed by Cauchy [58],

(6) $$n\left(\lambda \right)=A+\frac{B}{\lambda^{2}}+\frac{C}{\lambda^{4}}$$

where *A*, *B*, and *C* are constants. The Cauchy dispersion relation is simply an empirical fitting and bears no physical significance. A more significant dispersion model was given by Sellmeier [59],

(7) $$n^{2}\left(\lambda \right)=1+\Sigma_{i}\frac{A_{i}\lambda^{2}}{\lambda^{2}-\lambda_{i}^{2}}$$

where $A_{i}$ is a constant and subscript *i* denotes the multiple resonant wavelengths. The Sellmeier dispersion relation represents a more realistic model as whenever the electric field is impinged on a material, the electron clouds get disturbed by it, and the nuclei exert a restoring force, yielding the possibilities of multiple excitation. Since both of these Cauchy and Sellmeier relations are empirical, the concrete formulation for the dispersion relation was given by the Drude–Lorentz electronic theory [60]. This theory assumes that the electric field applied on an electron bound to the nucleus exerts Hooke’s force. Based on this model, it was found that the refractive index is associated with the oscillator strength ($C_{i}$) by [61],

(8) $$n^{2}\left(\omega \right)=1+\frac{Ne^{2}}{2\pi m}\Sigma_{i}\frac{C_{i}}{\omega_{i}^{2}-\omega^{2}}$$

where *N* is the particle density, *e* and *m* are the charge and mass of the electron, and $\omega_{i}$ and ω are the absorption and incident frequency, respectively. For a single oscillator, Wemple and DiDomenico introduced the semi-empirical relationship of the form [62,63],

(9) $$n^{2}\left(\nu \right)-1=\frac{E_{d}E_{o}}{E_{o}^{2}-{\left(h\nu \right)}^{2}}$$

where ν is the frequency, *h* is the Planck constant, $E_{o}$ is the single oscillator energy, and $E_{d}$ is the dispersion energy. The dispersion energy measures the average strength of interband optical transitions and is given by [62,63],

(10) $$E_{d}=\beta N_{c}N_{e}Z_{a}\left(eV\right),$$

where $N_{c}$ is the coordination number of the cation, $N_{e}$ is the effective number of valence electrons per anion, $Z_{a}$ is the formal charge of the anion, and β is a constant having the value 0.26 ± 0.04 eV for ionic compounds and 0.37 ± 0.05 eV for covalent compounds. Furthermore, based on the experimental data tested on several materials, it has been estimated empirically that the oscillator energy is related to the lowest energy gap by [62,63],

(11) $$E_{o}\approx 1.5E_{g}$$

where $E_{g}$ is the lowest direct band gap. Using the values of $N_{c}$, $N_{e}$, $Z_{a}$, and β for the perovskite structure in Equations (9)–(11), the Wemple and DiDomenico form for the static refractive index (n(0) or simply *n*) can be written as,

(12a) $$\begin{array}{cc} n & =\sqrt{1+\frac{16.64eV}{E_{g}}} \end{array}$$

(12b) $$\begin{array}{cc} n & =\sqrt{1+\frac{8.32eV}{E_{g}}} \end{array}$$

for oxide perovskites and halide perovskites, respectively.

These Equation (12a) and (12b) are based on the fact that the optoelectronic properties of perovskites are dependent on the ionic nature of the bonds or by simply treating perovskites as ionic solids. It is unambiguously known today that the octahedral cage in perovskites is formed by the heteropolar bonds (mixed ionic/covalent interactions) among the *B* cation and *X* anions, whereas the cation *A* shows electrostatic interaction with this cage [64,65]. A majority of the school of thought claims that the properties of perovskites are dependent on the octahedral cage built from the interaction of B−X ions, and the role of cation *A* is merely for the charge neutrality of the final stable structure [66,67,68]. In other words, the properties of perovskites evolve from the octahedral frame of B−X ions, and the cation *A* affects those properties by distorting this frame. Moreover, this claim can be justified by the Pauling rule [69], as in a multication system, cations with high valency and a small coordination number (CN) form polyhedra with the anion and the cation with low valency and a high coordination number (CN) adjusting their positions for final stability. The cation/anion ratio, as suggested by Pauling, determines the coordination number (CN), which in turn determines the structure of the polyhedra. In the case of perovskites, we take the cation/anion ratio as the average ratio for two cations, i.e., $\frac{\left(\frac{r_{A}}{r_{X}}+\frac{r_{B}}{r_{X}}\right)}{2}$, where $r_{A}$, $r_{B}$, and $r_{X}$ are the ionic radii of *A*, *B*, and *X* at CNs 12, 6, and 2, respectively [70].

Henceforth, one can paraphrase that the properties of perovskites are dependent on the octahedral cage created by B−X ions, and the adjustments of cation *A* for the final stable structure may lead to distorting this octahedral frame, which in turn influences these properties. Furthermore, the distortion of the octahedral cage induced by cation *A* may depend on the covalent nature of the B−X bond [71,72]. The greater the covalent B−X bond, the less is the distortion of the structure of $ABX_{3}$. Finally, incorporating the covalent nature of the B−X bond with the adjustment of *A* for the final stable structure, we propose the following modification of the Wemple and DiDomenico form,

(13a) $$\begin{array}{cc} n & =\sqrt{1+\frac{\left(r_{A}+r_{B}\right)}{r_{X}}\frac{11.84eV}{E_{g}}} \end{array}$$

(13b) $$\begin{array}{cc} n & =\sqrt{1+\frac{\left(r_{A}+r_{B}\right)}{r_{X}}\frac{5.92eV}{E_{g}}} \end{array}$$

for oxide perovskites and halide perovskites, respectively.

## 4. Results and Discussion

**Figure 1:**
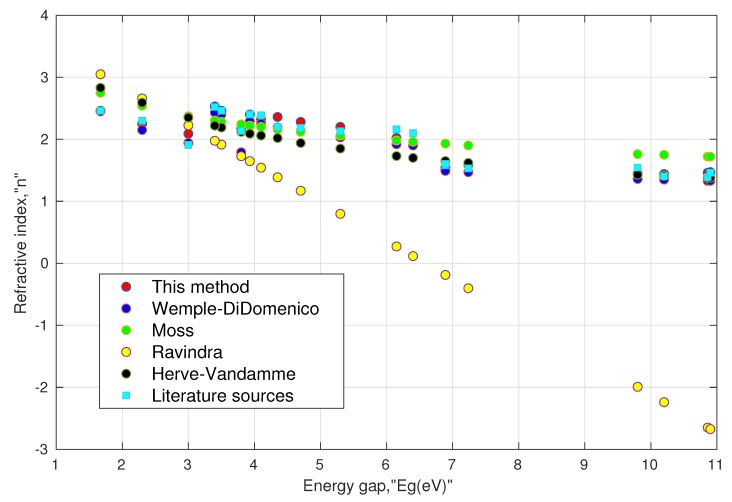
Comparison of various models with available literature data for perovskites, shown in Table 1.

I. Validity of the model: Based on Equation (13a) and (13b), we calculated the refractive index values of various oxide perovskites and halide perovskites. The results were then compared with the literature values obtained from various sources as shown in Table 1, and the resulting data are further plotted in Figure 1. One can notice that the computed results using (13a) and (13b) were in agreement with the corresponding literature values. These could further be compared with the values obtained from the Wemple–DiDomenico relation, Moss relation, Ravindra relation, and Herve–Vandamme relation. It must be noted that the reference values of the refractive index were not homogeneous in terms of the wavelength used, whereas the refractive index calculated by Equation (13a) and (13b) corresponded to the static or low-frequency values. Thus, this factor may contribute to some error during the comparison. Furthermore, we calculated the absolute accuracy error ($AAE=|X_{standard}-X_{calculated}|$) and mean absolute error (MAE=average(AAE)) to reckon the deviation of the proposed relation along with the other established relations with their corresponding refractive indices. It can be seen that the proposed model showed a mean absolute error of 0.07, which was the smallest of all the other established models.

**Figure 2:**
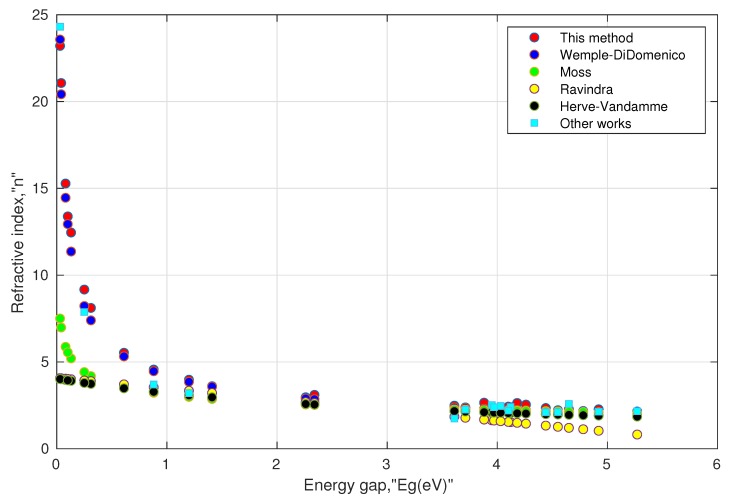
Simulated behavior of various models for oxide perovskites, shown in Table 2.

**Figure 3:**
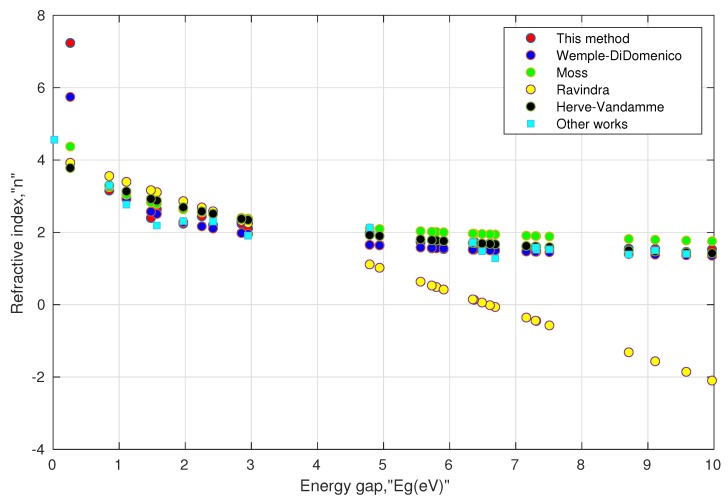
Simulated behavior of various models for halide perovskites, shown in Table 3.

II. Consistency of the model: As mentioned above, the experimental values of the direct energy gap and the refractive index values at low frequency are not frequently available for various perovskite materials. Moreover, the searched values were not as unique as they depended on the experimental methods used in the literature. Further, a few papers mentioned the phases and distortion of the structure before measuring the energy gap. Such inconsistency may impede the validity of the model. In order to remove such inconsistency, the energy gap values were taken from one common source [97] obtained from density functional theory (DFT) using the HSE (Heyd–Scuseria–Ernzerhof) functional. The results are shown in Table 2 and Table 3. We also included some available values of the refractive index in the last columns of these tables. They can serve as a reference and may be used with caution in comparing with other computed values. This is because of the fact that all the computed values of the refractive indices are a function of their respective energy gaps, and it is well known that the computed energy gaps using HSE underestimate the actual energy gaps [98]. Moreover, their structural phases may not be the same. For instance, in Table 3, the value of the refractive index (as shown by the last column) for orthorhombic (Pnma) $CsNaF_{3}$ is 4.56, whose energy gap is 0.019 eV as calculated by DFT using the GGA (generalized gradient approximation) functional, whereas the energy gap for cubic (Pm3m) $CsNaF_{3}$ is 0.26 eV using the HSE functional. Our model predicted a higher value of the refractive index for the energy gap close to zero, which was consistent for conducting materials. These results can be seen in Figure 2 and Figure 3. At higher energy gaps, this model converged with the Wemple–DiDomenico, Moss, and Herve–Vandamme models. One can notice that the prediction of the refractive index by the Ravindra relation showed negative values when the corresponding energy gaps were above 6.6 eV. This could be attributed to the non-parallelism in the trajectories of valence and conduction bands along the symmetry directions in perovskites [99,100,101]. Overall, the tabulated values indicated that Equations (13a) and (13b) were consistent enough for oxide perovskites and halide perovskites.

So far, we have seen that the proposed model not only predicts the refractive index of the perovskites with sufficient accuracy, but also shows a consistent behavioral pattern with some of the well-established models. However, it may not be appropriate to claim that the new formula is superior to these established models. First of all, the Wemple–DiDomenico model, Moss model, Ravindra model, and Herve–Vandamme model can be applied to all kinds of materials. However, the behavior of these models depends on various factors such as the types of bonds, the energy gaps, the nature of the materials like unary, binary, or ternary, etc. Secondly, all these models take a single argument, i.e., the smallest direct energy gap to compute the corresponding static refractive index. However, the proposed new formula could be applicable only to perovskites, and it takes two arguments: one is a structural parameter, quantified as the cation-anion ratio, and the other is the energy gap. Generally, perovskites are sensitive to an external stimulus, and therefore, the question of the stability of this structure is very crucial as many electro-optical properties depend on the evolution of the lattice. Therefore, one cannot solely depend on Wemple–DiDomenico model, Moss model, Ravindra model, and Herve–Vandamme model for perovskites as these models isolate structural reformation.

## 5. Conclusions

In summary, this study presented a new model to correlate the refractive index with the energy gap in perovskites. This model was tested on various oxide perovskites and halide perovskites, and the results obtained were in accord with some established models, as well as the literature values. All these models facilitated the calculation of the static refractive index based on the transition of valence electrons to the conduction band after absorbing the threshold photon energy, and henceforth, all these were discrete models. The efficacy of the proposed model was that it represented the correct picture of optical and electronic properties depending on the structural evolvement in perovskites. It took account of both structural distortion and the covalent nature of the B−X bond that were responsible for the fluctuations of the optoelectronic properties. Moreover, it is a well-established fact that the optoelectronic properties are susceptible to the structural reorganization in perovskites. Therefore, the more precise the measurement of the cation/anion ratio, the more accurate will be the correlation predicted by this model.

## Figures and Tables

**Table 1:** Comparison of refractive indices computed by various models with the literature values. AAE, absolute accuracy error.

Perovskite ${\mathrm{ABX}}_{3}$	Energy Gap ‘$E_{g}\left(eV\right)$’	Refractive Index ‘*n*’	This Method (Equation (13a) and (13b))	AAE	Wemple- DiDomenico Relation (Equation (12a) and (12b))	AAE	Moss Relation (Equation (1)	AAE	Ravindra Relation (Equation (4))	AAE	Herve- Vandamme Relation (Equation (5))	AAE
$SrTiO_{3}$	4.1 [73]	2.388 (632.8 nm) [73]	2.32	0.07	2.25	0.14	2.20	0.19	1.54	0.85	2.06	0.33
$SrSnO_{3}$	3.93 [74]	≈2.4 [75]	2.40	0.00	2.29	0.11	2.22	0.18	1.65	0.75	2.09	0.31
$KMgF_{3}$	10.2 [76]	1.404 (632.8 nm) [73]	1.44	0.04	1.35	0.05	1.75	0.35	N.V.	N.D.	1.41	0.01
$CaTiO_{3}$	3.5 [77]	≈2.46 [78]	2.46	0.00	2.40	0.06	2.28	0.18	1.91	0.55	2.19	0.27
$PbTiO_{3}$	3.4 [79]	2.52 [80]	2.53	0.01	2.43	0.09	2.30	0.22	1.98	0.54	2.22	0.3
$CsPbF_{3}$	3.8 [81]	2.134 (5.7 eV) [81]	2.17	0.04	1.79	0.34	2.24	0.11	1.73	0.40	2.12	0.01
$CsPbI_{3}$	1.67 [82]	2.46 (435 nm) [82]	2.46	0.00	2.45	0.01	2.75	0.29	3.05	0.59	2.83	0.37
$KTaO_{3}$	4.35 [83]	2.2 (632.8 nm) [73]	2.36	0.16	2.19	0.01	2.16	0.04	1.39	0.81	2.02	0.18
$CsPbBr_{3}$	2.3 [84]	≈2.3 (580 nm) [85]	2.25	0.05	2.15	0.15	2.54	0.24	2.66	0.36	2.59	0.29
$CsPbCl_{3}$	3.0 [84]	≈1.91 [86]	2.09	0.18	1.94	0.03	2.37	0.46	2.22	0.31	2.35	0.44
$LiTaO_{3}$	4.7 [87]	≈2.183 (632.8 nm) [73]	2.28	0.10	2.13	0.05	2.12	0.06	1.17	1.01	1.94	0.24
$BaZrO_{3}$	5.30 [88]	2.13 [89]	2.20	0.07	2.03	0.10	2.06	0.07	0.80	1.33	1.85	0.28
$SrZrO_{3}$	6.15 [88]	2.16 [90]	2.02	0.14	1.92	0.24	1.98	0.18	0.27	1.89	1.73	0.43
$CaZrO_{3}$	6.40 [88]	2.1 [91]	1.96	0.14	1.90	0.20	1.96	0.14	0.12	1.98	1.70	0.4
$KCaF_{3}$	10.86 [92]	1.388 (583.9 nm) [61]	1.46	0.07	1.33	0.06	1.72	0.33	N.V.	N.D.	1.38	0.01
$LiBaF_{3}$	9.8 [76]	1.544 (632.8 nm) [73]	1.45	0.09	1.36	0.18	1.76	0.22	N.V.	N.D.	1.43	0.11
$KZnF_{3}$	7.237 [93]	1.53 (583.9 nm) [61]	1.59	0.06	1.47	0.06	1.90	0.37	N.V.	N.D.	1.62	0.09
$RbCaF_{3}$	10.9 [94]	1.46 [95]	1.47	0.01	1.33	0.13	1.72	0.26	N.V.	N.D.	1.38	0.08
$CsCaCl_{3}$	6.89 [96]	1.58,1.603 (583.9 nm) [61,96]	1.54	0.04,0.06	1.49	0.09,0.11	1.93	0.35,0.33	N.V.	N.D.	1.65	0.07,0.05

N.V. represents a negative value *MAE*, 0.07, 0.11, 0.23, 0.87, 0.21. N.D. represents not defined.

**Table 2:** Energy gap of various oxide perovskites with their corresponding refractive indices computed from various models.

Oxide Perovskite ${\mathrm{ABO}}_{3}$	Energy Gap (HSE) ‘$E_{g}\left(eV\right)$’ [97]	$\frac{\left(\frac{r_{A}}{r_{X}}+\frac{r_{B}}{r_{X}}\right)}{2}$ [102]	This Method (Equation (13a))	Wemple– DiDomenico Relation (Equation (12a))	Moss Relation (Equation (1))	Ravindra Relation (Equation (4))	Herve– Vandamme Relation (Equation (5))	Other Works
$PbTiO_{3}$	3.95	0.78	2.38	2.28	2.21	1.64	2.09	≈2.52 [80]
$BaNbO_{3}$	0.31	0.85	8.11	7.39	4.18	3.89	3.73	−
$BaTiO_{3}$	4.12	0.82	2.39	2.24	2.19	1.53	2.05	≈2.4 [103]
$KTaO_{3}$	4.1	0.84	2.42	2.25	2.19	1.54	2.06	≈2.2 (632.8 nm) [73]
$NaNbO_{3}$	4.55	0.75	2.22	2.16	2.14	1.26	1.97	≈2.11 [104]
$SrFeO_{3}$	1.2	0.75	3.97	3.86	2.98	3.34	3.08	≈3.2 [105]
$SrVO_{3}$	0.04	0.75	21.07	20.42	6.98	4.06	4.00	−
$KTcO_{3}$	0.13	0.85	12.45	11.36	5.20	4.00	3.91	−
$TlIO_{3}$	4.26	0.98	2.54	2.21	2.17	1.44	2.02	−
$CsIO_{3}$	4.18	1.05	2.63	2.23	2.18	1.49	2.04	−
$CaFeO_{3}$	1.41	0.71	3.60	3.58	2.87	3.21	2.96	−
$BaZrO_{3}$	4.92	0.86	2.27	2.09	2.09	1.03	1.90	≈2.13 [89]
$BaSnO_{3}$	2.34	0.85	3.10	2.85	2.52	2.63	2.54	−
$KNbO_{3}$	4.44	0.84	2.35	2.18	2.15	1.33	1.99	≈2.1 [103]
$SrTiO_{3}$	3.97	0.76	2.35	2.28	2.21	1.62	2.08	≈2.388 (632.8 nm) [73]
$RbIO_{3}$	3.88	0.99	2.65	2.30	2.22	1.68	2.10	−
$BaBiO_{3}$	0.25	0.88	9.17	8.22	4.42	3.93	3.79	7.87 * [106]
$SrNbO_{3}$	0.08	0.79	15.28	14.46	5.87	4.03	3.96	−
$SrCrO_{3}$	0.61	0.76	5.53	5.32	3.53	3.71	3.48	−
$PbZrO_{3}$	4.65	0.82	2.27	2.14	2.13	1.20	1.95	≈2.58 [107]
$NaTaO_{3}$	4.78	0.75	2.17	2.12	2.11	1.12	1.93	−
$LaNiO_{3}$	0.03	0.68	23.21	23.57	7.50	4.07	4.01	24.31 ** [108]
$AgTaO_{3}$	3.61	0.79	2.48	2.37	2.26	1.85	2.17	≈1.736 [109]
$SrZrO_{3}$	5.27	0.80	2.14	2.04	2.06	0.82	1.85	≈2.16 [90]
$LaCrO_{3}$	3.71	0.73	2.38	2.34	2.25	1.78	2.14	≈2.25 [110]
$LaMnO_{3}$	2.26	0.74	2.96	2.89	2.55	2.68	2.58	−
$CaTiO_{3}$	4.03	0.74	2.32	2.26	2.20	1.59	2.07	≈2.46 [78]
$LaTiO_{3}$	0.1	0.75	13.38	12.94	5.55	4.02	3.94	−
$SrCoO_{3}$	0.88	0.74	4.56	4.46	3.22	3.54	3.28	≈3.7 [105]

* Estimate based on reflectivity of 0.6 at 0 K; ** estimate based on reflectivity of 0.85 at 0 K.

**Table 3:** Energy gap of various halide perovskites with their corresponding refractive indices computed from various models.

Halide Perovskite ${\mathrm{ABX}}_{3}$	Energy Gap (HSE) ‘$E_{g}\left(eV\right)$’ [97]	$\frac{\left(\frac{r_{A}}{r_{X}}+\frac{r_{B}}{r_{X}}\right)}{2}$ [102]	This Method (Equation (13b))	Wemple– DiDomenico Relation (Equation (12b))	Moss Relation (Equation (1))	Ravindra Relation (Equation (4))	Herve–Vandamme Relation (Equation (5))	Other Works
$TlZnF_{3}$	5.8	0.95	1.71	1.56	2.01	0.49	1.78	−
$CsSnCl_{3}$	1.57	0.79	2.64	2.51	2.79	3.11	2.88	≈2.19 [111]
$CsGeI_{3}$	1.48	0.59	2.40	2.57	2.83	3.17	2.92	−
$KMgF_{3}$	9.58	0.92	1.46	1.37	1.77	N.V.	1.44	≈1.404 (632.8 nm) [73]
$KCdF_{3}$	6.69	1.01	1.67	1.50	1.94	N.V.	1.67	≈ 1.28 [96]
$RbZnF_{3}$	7.16	0.96	1.61	1.47	1.91	N.V.	1.62	−
$RbCaF_{3}$	9.11	1.06	1.54	1.38	1.80	N.V.	1.47	≈1.5 [112]
$RbCdF_{3}$	6.61	1.04	1.69	1.50	1.95	N.V.	1.68	−
$RbCaCl_{3}$	7.31	0.76	1.49	1.46	1.90	N.V.	1.61	≈1.52 [112]
$RbMnF_{3}$	6.49	0.99	1.68	1.51	1.96	0.06	1.69	≈1.478 [61]
$CsNaF_{3}$	0.26	1.13	7.24	5.74	4.37	3.92	3.78	4.56 * [90,97]
$TlCdBr_{3}$	5.91	0.68	1.54	1.55	2.00	0.42	1.76	−
$RbNiF_{3}$	5.56	0.94	1.73	1.58	2.03	0.64	1.81	−
$CsCaF_{3}$	9.97	1.12	1.53	1.35	1.76	N.V.	1.42	−
$CsPbF_{3}$	4.79	1.19	1.99	1.65	2.11	1.11	1.93	≈2.134 (5.7 eV) [81]
$CsSnBr_{3}$	1.11	0.72	2.96	2.91	3.04	3.39	3.13	≈2.769 [111]
$CsCdF_{3}$	6.37	1.10	1.74	1.52	1.96	0.13	1.70	−
$KZnF_{3}$	7.51	0.93	1.57	1.45	1.88	N.V.	1.59	≈1.53 (583.9 nm) [61]
$CsCaCl_{3}$	7.3	0.80	1.52	1.46	1.90	N.V.	1.61	≈1.58, 1.603 (583.9 nm) [61,96]
$CsGeBr_{3}$	1.97	0.67	2.24	2.28	2.63	2.86	2.69	≈2.31 [113]
$CsPbBr_{3}$	2.42	0.79	2.20	2.11	2.50	2.58	2.52	≈ 2.3 (580 nm) [85]
$CsCaBr_{3}$	6.35	0.74	1.54	1.52	1.97	0.15	1.71	≈1.72 [90]
$CsPbCl_{3}$	2.95	0.86	2.11	1.95	2.38	2.25	2.34	≈1.91 [86]
$CsSnI_{3}$	0.85	0.64	3.15	3.28	3.25	3.56	3.30	≈3.3 [111]
$KCaF_{3}$	8.71	1.03	1.55	1.40	1.82	N.V.	1.50	≈ 1.388 (583.9 nm) [61]
$KMnCl_{3}$	5.73	0.69	1.56	1.56	2.02	0.53	1.78	−
$TlFeF_{3}$	2.85	0.96	2.24	1.98	2.40	2.32	2.37	−
$LiSnCl_{3}$	4.94	0.72	1.65	1.64	2.09	1.02	1.90	−
$TlCuF_{3}$	2.25	0.95	2.44	2.17	2.55	2.69	2.58	−

N.V. represents a negative value; * orthorhombic lattice, $E_{g}=0.019$ eV.
